# Sacha Inchi Oil Press-Cake Protein Hydrolysates Exhibit Anti-Hyperuricemic Activity via Attenuating Renal Damage and Regulating Gut Microbiota

**DOI:** 10.3390/foods11162534

**Published:** 2022-08-22

**Authors:** Kun Wang, Shanshan Wu, Pan Li, Nan Xiao, Jiamin Wen, Jinming Lin, Siming Lu, Xin Cai, Yanan Xu, Bing Du

**Affiliations:** College of Food Science, South China Agricultural University, Guangzhou 510642, China

**Keywords:** sacha inchi oil press-cake protein hydrolyzates, hyperuricemia, renal injury, gut microbiota

## Abstract

The incidence of hyperuricemia has increased globally due to changes in dietary habits. The sacha inchi oil press-cake is generally discarded, resulting in the waste of resources and adverse environmental impact. For the purpose of developing sacha inchi oil press-cake and identifying natural components with anti-hyperuricemic activities, we systemically investigated the underlying mechanisms of sacha inchi oil press-cake protein hydrolysates (SISH) in the hyperuricemic rat model. SISH was obtained from sacha inchi oil press-cake proteins after trypsin treatment, and 24 peptides with small molecular weight (<1000 Da) were identified. The results of animal experiments showed that SISH significantly decreased the serum uric acid (UA) level by inhibiting the xanthine oxidase (XOD) activity and regulating the gene expression related to UA production and catabolism in hyperuricemia rats, such as Xdh and Hsh. In addition, SISH attenuated the renal damage and reduced the gene expression related to inflammation (Tlr4, Map3k8, Pik3cg, Pik3ap1, Ikbke, and Nlrp3), especially Tlr4, which has been considered a receptor of UA. Notably, SISH reversed high purine-induced gut microbiota dysbiosis, particularly by enhancing the relative abundance of butyric acid-producing bacteria (unidentified_*Ruminococcaceae*, *Oscillibacter*, *Ruminiclostridium*, *Intestinimonas*). This research provided new insights into the treatment of hyperuricemia.

## 1. Introduction

Hyperuricemia has become prevalent due to changes in dietary habits [1,2]. It is a leading cause of gout [3,4] and a risk factor for hypertension [5], obesity [6], cardiovascular disease [7], and renal failure [8]. Hyperuricemia is characterized by an increase in uric acid (UA) levels in serum due to the imbalance between the blood UA generation and excretion [9]. UA is one product in the purine metabolism pathway. Xanthine oxidase (XOD), a critical enzyme in the purine catabolic pathway, catalyzes the oxidation and conversion of hypoxanthine and xanthine to UA [10]. In in vivo settings, kidneys are responsible for most UA excretion, while approximately 25% of UA is excreted into the intestines and further metabolized by gut microbiota [9]. In fact, a recent report indicated that gut microbiota could serve as a candidate intervention target to relieve hyperuricemia pathogenesis [9]. Therefore, both kidney health and gut microbiota integrity are critical in hyperuricemia prevention. At present, three categories of medicines are commonly used to treat hyperuricemia: inhibitors of xanthine oxidase, uricosuric agents, and recombinant uricases [11,12]. However, these drugs have unfavorable side effects, such as serious allergic reactions and rashes resulting from allopurinol [13]. Consequently, it is critical to identify more effective alternatives to manage hyperuricemia with fewer side effects.

Protein hydrolysates are more digestible than protein, releasing more bioactive peptides and exhibiting a variety of biological functions, such as anticancer and immunoregulation [14]. Recently, it has been reported that certain protein hydrolysates exhibited anti-hyperuricemic activity. Whey protein hydrolysates lessened renal dysfunction caused by hyperuricemia by restraining the production of UA and promoting the excretion of UA [15]. Bonito protein hydrolysates significantly reduce the serum UA level, inhibit the XOD enzyme activity, and alleviate renal damage in hyperuricemic rats [16]. In addition, walnut protein hydrolysate, tuna protein hydrolysates, and kidney bean protein hydrolysates exhibited anti-hyperuricemic activity [17,18,19]. Therefore, protein hydrolysates are reasonable resources to treat hyperuricemia. However, despite these previous studies that documented the anti-hyperuricemic activity of protein hydrolysates, a systemic and comprehensive investigation is needed to mechanistically address several essential unknowns. For instance, the major components and compositions of these hydrolysates need to be clarified, and the physiological impacts of protein hydrolysates on the kidney and gut biology and physiology demand specifications.

Sacha inchi (*Plukenetia volubilis* L.) is a plant belonging to the Euphorbiaceae family native to the Amazon Rainforest in Peru [20]. Sacha inchi kernels serve as an enriched source of various nutrients, especially in the forms of oils (35~65%) and proteins (25~30%) [21]. Sacha inchi kernels are mainly used for oil extraction, with the press-cake as a by-product. Although sacha inchi oil press-cake is rich in high-quality proteins (47~59%), it has been generally ignored, causing a massive waste of protein resources [22]. In addition, improper handling of the press-cake can readily result in environmental pollution. As reported, green tea residue contained 18~20% protein, and its hydrolysates showed various biological activity, including antioxidant and anti-hypertensive activity [23]. Sacha inchi oil press-cake is also a good source of protein hydrolysates with various biological activities [24,25]. Consistently, upon hydrolyzation of the sacha inchi oil press-cake by calotropis proteases and crude papain, the obtained protein hydrolysates possess substantial antioxidant properties [25]. In line with these findings, hydrolyzation of the sacha inchi oil press-cake by alcalase, neutrase, and flavourzyme yields protein hydrolysates that have antioxidant activities, as well [24]. Therefore, sacha inchi oil press-cake protein hydrolysates may be an effective way to address resource waste. However, the therapeutic value of the sacha inchi oil press-cake protein hydrolysates on hyperuricemia has not been defined yet.

Given the above considerations, there are multiple advantages to further developing sacha inchi oil press-cake to bring gains in the economy, environment, and the sacha inchi industry. In addition, it is also very important to explore more effective alternatives to treat and prevent hyperuricemia. Therefore, the aim of this study was to find the sacha inchi oil press-cake protein hydrolysates (SISH) with anti-hyperuricemic activity. Additionally, 16S rRNA gene sequencing, UPLC-Q-TOF/MS-based metabolomics, and RNA-Seq transcriptomics were used to determine the anti-hyperuricemic mechanisms underlying SISH. This study provides novel insights into the utilization of SISH and the treatment of hyperuricemia.

## 2. Materials and Methods

### 2.1. Preparation and Characterization of SISH

About 5 kg of defatted and squeezed sacha inchi seed flour was provided by the Pure U-Multitude Biological Resources Co., Ltd. (Yunnan Province, China). The sacha inchi seed flour was soaked in water (1:25, *w*/*v*) for 3 h. After that, the solution was hydrolyzed with 5% trypsin at 55 °C for 6 h, and the enzyme was inactivated by heating at 90 °C for 15 min [26]. Upon centrifugation (4000× *g*, 10 min), the supernatant was passed through an ultrafiltration membrane (3000 Da). The permeated solvent was then concentrated by evaporation and lyophilized to obtain SISH.

The amino acid analysis of SISH was conducted similarly as previously described [27]. The Shimadzu LC-20A HPLC system (Shimadzu, Japan) was used to measure the molecular weight distribution of SISH content according to the method described by Li et al. [28]. The peptide sequence of SISH was similarly identified using high-performance liquid chromatography-mass spectrometry (LC/MS-MS) [29].

### 2.2. Animals and Experimental Design

All animal experiments were conducted in the Guangdong Medical Laboratory Animal Center (Approval No: SCXY(Yue) 2018-0137) in accordance with Chinese laws and regulations on the use and care of laboratory animals. A total of 36 male Sprague Dawley (SD) rats were provided by the Medical Experimental Animal Center of Guangdong Province and housed in standard laboratory cages (temperature, 22~26 °C; humidity, 40~60%) with 10 h light/14 h dark cycle.

After adaptive feeding for 7 days, the rats were randomly divided into the blank control (CD) group (*n* = 9) and the model groups (*n* = 27). The model groups were orally gavaged with adenine (200 mg/kg BW) and injected intraperitoneally with potassium oxonate (100 mg/kg BW), while the CD group was fed with a normal diet. After feeding for 10 days, eyelid blood was collected to determine the serum UA level. UA levels above 110 μmoL/L were considered to be qualified as hyperuricemic in the rat model [16]. Then, the administration of adenine and potassium oxonate was halted, and the model group rats were randomly divided into three groups (*n* = 9 each), the hyperuricaemic model group (MG), the SISH treatment group (SISH), and the allopurinol treatment group (DG). Rats in the SISH and DG groups were intragastrically administered SISH (0.42 g/kg BW) and allopurinol (10 mg/kg BW), respectively, while the CD and MG groups were given the same volume of 0.9% saline. After SISH treatment for 4 weeks, the blood, liver, kidney, and colon contents were sampled and stored at −80 °C until use.

### 2.3. Biochemical Determination

XOD activity and the levels of UA, creatinine (Cr), and blood urea nitrogen (BUN) in the rat serum were determined by commercial detection kits (Jiancheng Bioengineering Institute, Nanjing, China) according to instructions of the manufacturer. The liver tissues were homogenized and centrifuged (3000 r/min, 10 min) at 4 °C, and XOD activity in the supernatant was then determined by the kit accordingly.

### 2.4. Histology

The kidney samples were stained with hematoxylin-eosin (HE) and examined by light microscopy. Scoring the renal tubule and interstitial injury was set up based on the following criteria: 0 points = no significant lesion; 1 point = the range of lesions does not exceed 1/4 area; 2 points = the range of lesions does not exceed 1/2 area; 3 points = the lesion range does not exceed 3/4 area; 4 points = the lesion range exceeds 3/4 area.

### 2.5. 16S rRNA Gene Amplification and Sequencing

The colon contents samples of each rat (*n* = 6) were collected aseptically for 16S rRNA gene sequencing analysis. According to the manufacturer’s recommendation, genomic DNA from the colonic content was extracted through a fecal DNA isolation kit (MoBio Laboratories, Carlsbad, CA, USA). The primers 515F (5′-GTGCCAGCMGCCGCGGTAA-3′) and 806R (5′-GGACTACHVGGGTWTCTAAT-3′) were used to amplify the V4 region of 16S rRNA genes. The resulting PCR products were purified with GeneJETTMGel Extraction Kit (Thermo Scientific, Waltham, MA, USA). According to the manufacturer’s recommendation, the Ion Plus Fragment Library Kit 48 rxns (Thermo Scientific) was used to generate the sequencing library. The sequencing test was performed by the TNovogene Bioinformatics Technology Co., Ltd. (Tianjin, China) using the Illumina MiSeq sequencing platform.

In order to obtain high-quality clean reads, the raw reads were trimmed and filtered under standard operations based on Cutadapt (V1.9.1, http://cutadapt.readthedocs.io/en/stable/, accessed on 13 January 2021). The operational taxonomic units (OTUs) with 97% sequence similarity were obtained using the Uparse software (Version 7.0.1001; Edgar, Robert C; Belvedere Tiburon, CA, USA). With the Mothur algorithm, the Silva Database (https://www.arb-silva.de/, accessed on 13 January 2021) was used to annotate the taxonomic information of each representative sequence. The alpha diversity and beta diversity of the gut microbiota were calculated by QIIME (Version 1.7.0; Rob Knight; Fort Collins, CO, USA). Principal Coordinate Analysis (PCoA) was performed with the WGCNA package, stat packages, and ggplot2 package in the R software (Version 2.15.3; Mathsoft Company; Cambridge, MA, USA). Anosim and multi-response permutation procedure (MRPP) analyses were conducted with QIIME. The Galaxy platform was used for linear discriminant analysis effect size (LEfSe) analysis based on *p* < 0.05 and LDA score > 3 [30].

### 2.6. Serum Metabolomic Analysis

A total of 100 μL of serum samples were mixed with 400 μL of prechilled methanol. All samples were centrifugated at 15,000 rpm for 5 min, and the supernatant was diluted to a final concentration of 60% methanol. After filtration with a 0.22 μm filter, samples were centrifuged at 15,000× *g* for 10 min. Each sample was subjected to ultra-high performance liquid chromatography analysis in combination with quadrupole time-of-flight mass spectrometry (UHPLC-MS/MS) (Thermo Fisher). Samples were injected onto a Hyperil Gold column (100 × 2.1 mm, 1.9 μm) at a flow rate of 0.2 mL/min by a 16 min linear gradient. Eluent A (aqueous solution containing 0.1% fomic acid) and eluent B (methanol) were used for the positive polar mode, while eluent A (5 mM ammonium acetate, pH 9.0) and eluent B (methanol) were used for the negative polar mode. The solvent gradient was set as follows: 2% B, 1.5 min; 2–100% B, 12.0 min; 100% B, 14.0 min; 100–2% B, 14.1 min; 2% B, 16 min. Q ExActive HF-X mass spectrometer was operated in positive/negative polarity mode with the following parameters: spray voltage of 3.2 kV, capillary temperature of 320 °C, sheath gas flow rate of 35 arb, and auxiliary gas flow rate of 10 arb. Compound Discoverer 3.0 (Thermo Fisher) was used to analyze the original data files generated by the UHPLC–MS/MS, and peak alignment, peak calling, and quantitation were performed for each metabolite. In order to obtain accurate qualitative and relative quantitative results, peaks were aligned with mzCloud (https://www.mzcloud.org/, accessed on 21 December 2020) and ChemSpider (http://www.chemspider.com/, accessed on 21 December 2020) databases. Multivariate analyses, including principal component analysis (PCA) and Orthogonal partial least-squares discriminant analysis (OPLS-DA), were performed using the SIMCA-P 11.0 software (Umetrics AB, Umea, Sweden). Metabolites with significant differences between groups were obtained under variable influence on projection (VIP) > 1, and *p*-value < 0.05 based on the peak areas.

### 2.7. Renal Transcriptome Analysis

Total RNA was isolated from kidneys in compliance with the protocol of TRIzol reagent (Beyotime, Shanghai, China). The integrity and quality of total RNA were evaluated with the RNA Nano 6000 Assay Kit of the Bioanalyzer 2100 system (Agilent Technologies, Palo Alto, CA, USA). Then, 3 μg of total RNA for each sample was used as the input material for the RNA sample preparations. Based on the manufacturer’s recommendations and index codes, TruSeq RNA Sample Preparation Kit was applied to generate the sequencing libraries, which were then sequenced on the Illumina HiSeq 2500 platform by the Novogene Bioinformatics Technology Co., Ltd. (Tianjin, China).

Index of the reference genome was established, followed by alignment using Hisat2 (Version2.0.5; Daehwan Kim; Missouri City, TX, USA) with the paired-end clean reads. The relative expression of each gene was normalized and inferred using fragments per kilobase of transcript per million mapped reads (FPKM). Differential expressions with statistical analysis were calculated using the software DESeq2 R package (Version 1.16.1; Michael I Love; Heidelberg, Germany), and the threshold was set as Fold Change > 1.5 and *p*-value < 0.05. Finally, the Gene Ontology (GO) program was applied to annotate the unigenes and analyze the pathway enrichment for differential expression genes (DEGs) [31].

### 2.8. Correlation Analysis and Statistical Analysis

The statistical analysis of quantitative data was conducted with the one-way variance analysis (one-way ANOVA) followed by Duncan’s multiple comparisons test using the SPSS 16.0. Spearman’s correlation coefficients of gut microbiota, DEGs, and metabolites were calculated with R software (v3.2.1; Mathsoft Company) and visualized with the Complex Heatmap package in R software (v3.2.1). All data are expressed as means ± SD. 

## 3. Results

### 3.1. Identification of SISH Composition

SISH was obtained from sacha inchi oil press-cake upon trypsin treatment, and the molecular weight distribution and amino acid composition of SISH were determined (Table 1). The results indicated that most of the peptides in SISH were oligopeptides with a molecular weight of less than 1000 Da (90.70%). In addition, SISH contained 17 kinds of amino acids in total, mainly including glutamic acid (16.7%), aspartic acid (12.6%), and arginine (10.8%). Among them, the essential amino acid content was in the range of 30%, and the hydrophobic amino acid content was 24.9%. De novo sequencing was performed on the collected high-resolution mass spectrometry dataset, and 24 polypeptides were identified based on scores, peak areas, fragment ion error distribution, and other factors (Table 2). MVVKK, KVVL, RLLVWELER, WLPDVK, and TVLLPR were identified as the most abundant peptides in SISH. 

### 3.2. SISH Attenuated Hyperuricemia

**Figure 1:**
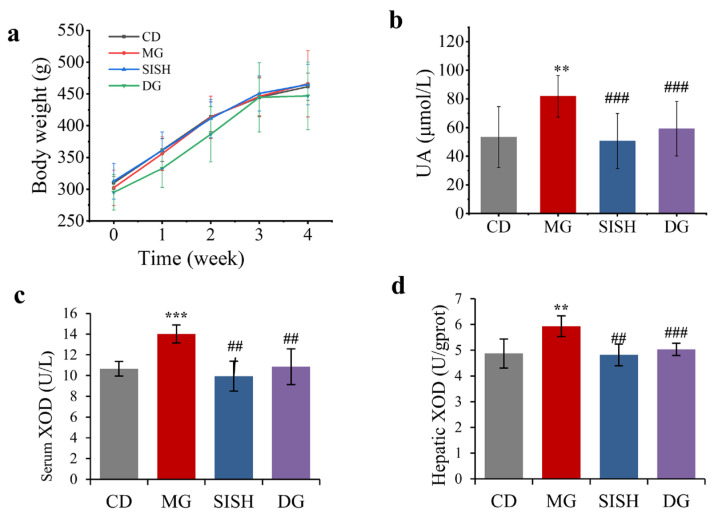
Evaluation of SISH anti-hyperuricemic activities in hyperuricemic rats. (**a**) Body weight. (**b**) Serum uric acid (UA) levels. (**c**) Serum xanthine oxidase (XOD) activity. (**d**) Hepatic XOD activity. All data are expressed as means ± SD. ** *p* < 0.01, and *** *p* < 0.001 vs. the CD group; ^##^ *p* < 0.01, and ^###^ *p* < 0.001 vs. the MG group.

Established SD rat models were used to evaluate the anti-hyperuricemia activity of SISH, and elevated serum UA level was monitored as an indicator, which is a common clinical observation in patients with hyperuricemia. After the initial 10 days of high purine diet feeding, the levels of serum UA were above 110 μmoL/L in the MG, SISH, and DG groups, a significant increase (*p* < 0.05) as compared with the CD group, demonstrating the success in the establishment of the hyperuricemic rat model (Supplementary Figure S1). After four weeks of indicated SISH treatments, there were no significant differences in body weight among the four test groups (*p* > 0.05) (Figure 1a). However, the serum UA of hyperuricemic rats after SISH treatment significantly decreased by 38.56% compared to the MG group (Figure 1b). As reported, XOD is highly expressed in the liver, and an increase in XOD activity would enhance UA production [32]. Therefore, we next examined the effect of SISH treatment on the XOD activity in hyperuricemic rats. As compared with the CD group, XOD activity in serum and hepatic was significantly increased in the MG group (*p* < 0.001), and its activity was reversed to normal levels upon SISH supplementation (*p* < 0.01) (Figure 1c,d). These results indicated that SISH could effectively rescue the induced hyperuricemia by inhibiting XOD activity.

### 3.3. SISH Attenuated Renal Damage in Hyperuricemic Rats

**Figure 2:**
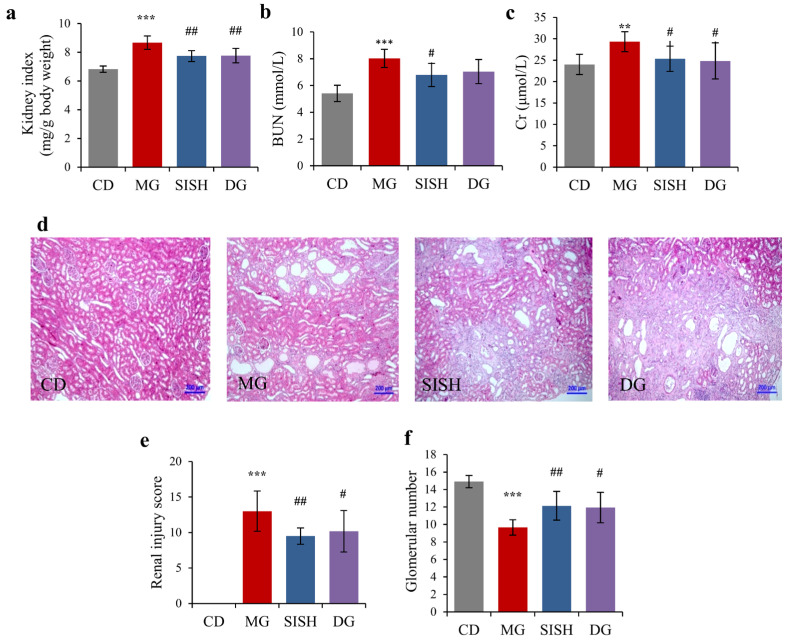
The effects of SISH on renal damage in hyperuricemic rats. (**a**) The kidney indexes. (**b**) Blood urea nitrogen (BUN) levels. (**c**) Serum creatinine (Cr) levels. (**d**) Photomicrographs of representative sections of kidneys. (**e**) Renal injury score. (**f**) Glomerular number in each group. All data are expressed as means ± SD. ** *p* < 0.01, and *** *p* < 0.001 vs. the CD group; ^#^ *p* < 0.05, and ^##^ *p* < 0.01 vs. the MG group.

Hyperuricemia would increase the risk of renal injury, and thus we needed to examine the effects of SISH on the rat kidney. As shown, SISH supplement indeed profoundly reduced the kidney index as compared with the MG group (Figure 2a). The BUN and Cr, as the indexes of kidney functions, were also evaluated in hyperuricemic rats (Figure 2b,c). The levels of BUN and Cr were substantially increased in the MG group, indicating that the renal functions of hyperuricemia rats were impaired. However, both the BUN and Cr levels were markedly decreased by 12.12% and 13.63% upon SISH treatment (*p* < 0.05). These results showed that SISH could ameliorate renal damage in hyperuricemic rats. Histologically, the sections of kidneys from the CD group showed normal architecture with intact glomerulus and renal tubules. However, in contrast to the normal group, glomerular atrophy, renal tubular dilation, and inflammatory cell infiltration were present in the MG group, suggesting that hyperuricemic rats had obvious renal damage (Figure 2d). Nevertheless, renal lesions were alleviated upon SISH treatment, which was manifested by a moderate decrease in glomerular volume, mild tubules dilation, and mild inflammatory cell infiltration in the interstitium. Simultaneously, injury scores in the SISH group were also significantly lower than that in the MG group (Figure 2e), and the glomerular counts were similarly reversed (Figure 2f). These results showed that SISH treatment effectively relieved the renal damage in hyperuricemic rats.

### 3.4. SISH Reshaped the Gut Microbiota Community in Hyperuricemic Rats 

**Figure 3:**
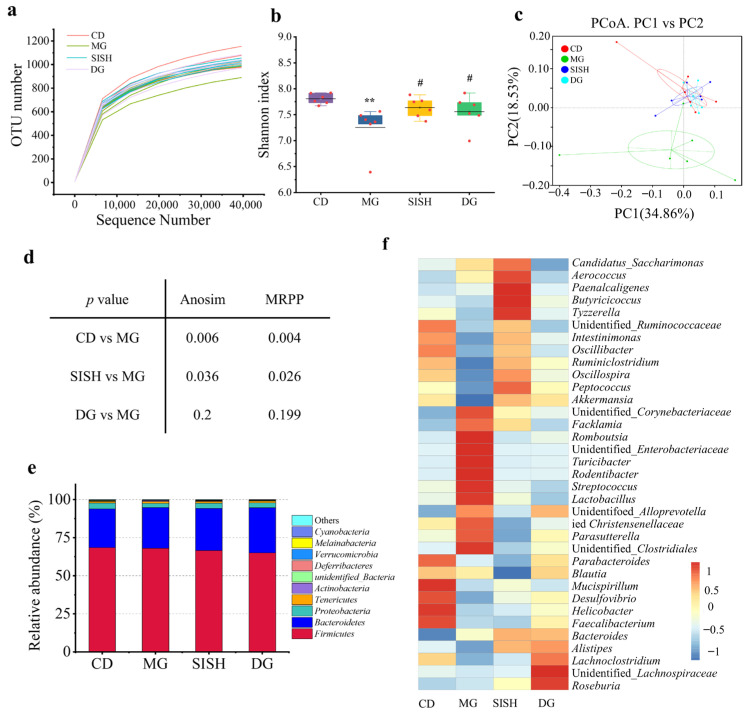
The effects of SISH on the diversity and integrity of the gut microbiota in hyperuricemic rats. (**a**) Rarefaction curves. (**b**) Shannon index. (**c**) Principal coordinate analysis (PCoA). (**d**) Anosim and multi-response permutation procedure (MRPP) analyses. (**e**) Relative abundance at phylum levels. (**f**) The heatmap analysis at genus levels. All data are expressed as means ± SD. ** *p* < 0.01 vs. the CD group; ^#^ *p* < 0.05 vs. the MG group.

The gut microbiota alteration was analyzed by 16S rRNA gene sequencing. A total of 2,021,638 clean reads were generated based on sequencing the V4 region of 16S rRNA genes, and each colonic sample produced an average of 84,234 ± 4205 clean reads. The rarefaction curves were close to the saturation plateau (Figure 3a), indicating that the sequencing depth covered rare new phylotypes and represented most of the accessible microbiota. The Shannon index that indicates the diversity of microbial communities [33] was significantly decreased in the MG group as compared to that of the CD group (*p* < 0.001). Upon SISH and allopurinol treatments, the Shannon index was significantly increased in the SISH and DG groups as compared with the MG group (Figure 3b). Therefore, SISH clearly increased the diversity of gut microbiota in hyperuricemic rats. Meanwhile, PCoA analysis also showed that the community composition of the MG group was separated from the other three groups (Figure 3c). Anosim and MRPP algorithms further confirmed significant differences (*p* < 0.05) between the MG groups versus the CD, SISH, and DG groups (Figure 3d).

Taxonomic profiling was also used to define SISH effects on the gut microbiota composition in hyperuricemic rats (Figure 3e). As shown, *Firmicutes*, *Bacteroidetes*, *Proteobacteria*, and *Tenericutes* were the dominant phylum in all test groups (Figure 3e). There was no significant difference among groups at the phylum levels. Nevertheless, heatmap analysis showed a dramatic alteration of gut microbiota distribution at the genus level (Figure 3f). Compared with the CD group, *Romboutsia* (1.64% vs. 6.71%) and unidentified_*Clostridiales* (0.42% vs. 0.80%) were markedly increased, while unidentified_*Ruminococcaceae* (9.63% vs. 6.16%), *Ruminiclostridium* (1.54% vs. 0.66%), *Oscillibacter* (1.92% vs. 0.88%), and *Intestinimonas* (1.01% vs. 0.52%) were significantly decreased in the MG group. Of importance, SISH intervention substantially rescued the relative abundance of those intestinal florae in hyperuricemic rats (Figure 3f, Supplementary Figure S2). Specifically, SISH enhanced the relative abundance of probiotics such as *Akkermansia* and *Alistipes* and reduced the *Streptococcus* (XOD-producing bacteria) population [10]. Taken together, these results evidenced that SISH could modulate the gut microbial organization and enhance the microbial diversity in hyperuricemic rats.

### 3.5. SISH Intervention Altered Serum Metabolome in Hyperuricemic Rats

**Figure 4:**
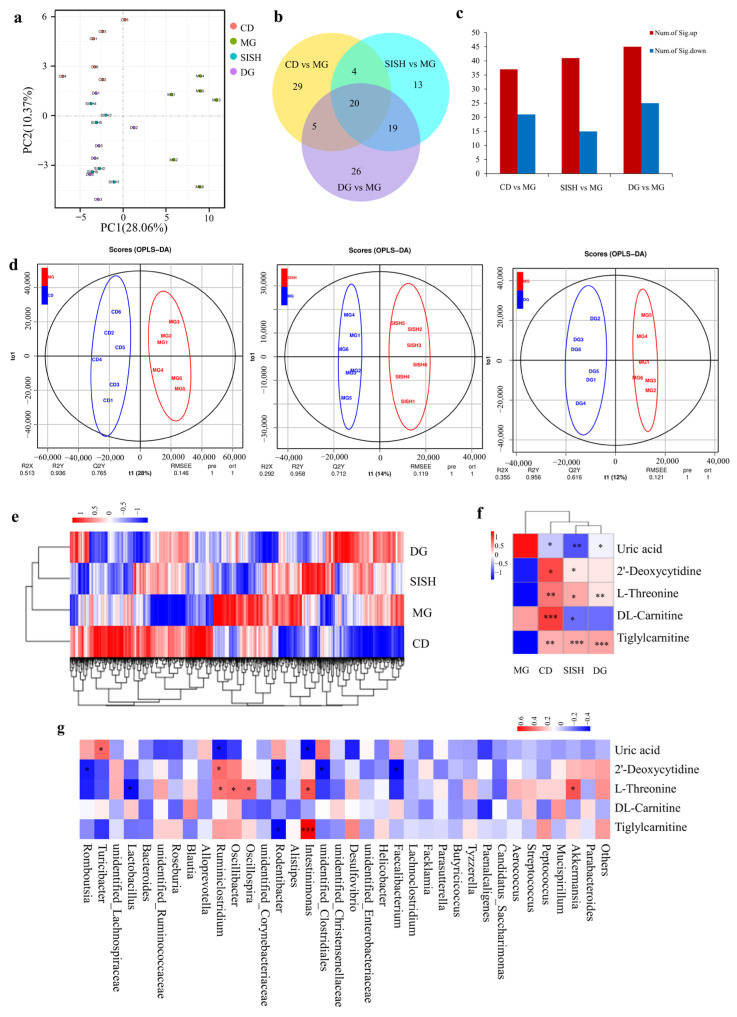
The impacts of SISH on the serum metabolome of hyperuricemic rats. (**a**) Principal component analysis (PCA) score plot. (**b**) Venn diagram. (**c**) The numbers of differential metabolites. (**d**) The Orthogonal partial least-squares discriminant analysis (OPLS-DA) scores plot. (**e**) A heatmap of differential metabolites potentially associated with hyperuricemia. (**f**) A heatmap of differential metabolites potentially associated with hyperuricemia disease for each treatment. (**g**) Spearman correlation analysis between the metabolites and the gut microbiota. Note: * *p* < 0.05, ** *p* < 0.01, and *** *p* < 0.01.

The effects of SISH on serum metabolome in hyperuricemia rats were next determined by the UHPLC-MS/MS analyses. PCA score plotting showed a clear separation trend between the CD and the MG groups, with similar separation trends being observed between the SISH and the MG groups and between the DG and the MG groups (Figure 4a), respectively. In comparison with that of the MG group, a total of 58, 56, and 70 metabolites were significantly changed in the CD, the SISH, and the DG groups, respectively (Figure 4b,c). OPLS-DA analysis further showed that there was a distinct classification in the metabolite profiles between the MG and the SISH groups (Figure 4d). In addition, cluster analyses revealed a distinct separation of serum metabolites between the MG and the SISH groups (Figure 4e). We also found that UA levels were significantly increased, while 2′-deoxycytidine levels were decreased in the MG group. Other metabolites related to amino acid and energy metabolism, such as L-threonine, DL-carnitine, and tigliylcarnitine, were also substantially reduced in the MG group, indicating that hyperuricemia is extensively linked to amino acid and energy metabolic dysfunction [34,35]. In any case, upon SISH treatment, levels of the above five metabolites were all pronouncedly restored. In comparison, allopurinol treatment rescued the levels of UA, L-threonine, and tigliylcarnitine only (Figure 4f, Supplementary Table S1).

We further performed Spearman correlation analysis to visualize an association between the gut microbiota and metabolome (Figure 4g). As shown, UA was positively (*p* < 0.05) correlated with the gut genus of *Turicibacter* while negatively (*p* < 0.05) correlated with the gut genus of *Ruminiclostridium* and *Intestinimonas*. 2′-deoxycytidine was positively (*p* < 0.05) correlated with *Ruminiclostridium* while negatively (*p* < 0.05) correlated with *Romboutsia*, unidentified_*Clostridiales*, *Rodentibacter,* and *Faecalibacterium*. Metabolite of L-threonine was positively (*p* < 0.05) correlated with the gut genu of *Ruminiclostridium*, but negatively (*p* < 0.05) correlated with the gut genus of *Romboutsia*, unidentified_*Clostridiales*, *Rodentibacter,* and *Faecalibacterium*. Overall, these results delineated the impacts of SISH on the serum metabolome in hyperuricemic rats.

### 3.6. Identification of the Potential SISH Target Genes and Signaling Pathways Involved in Hyperuricemic Rats

**Figure 5:**
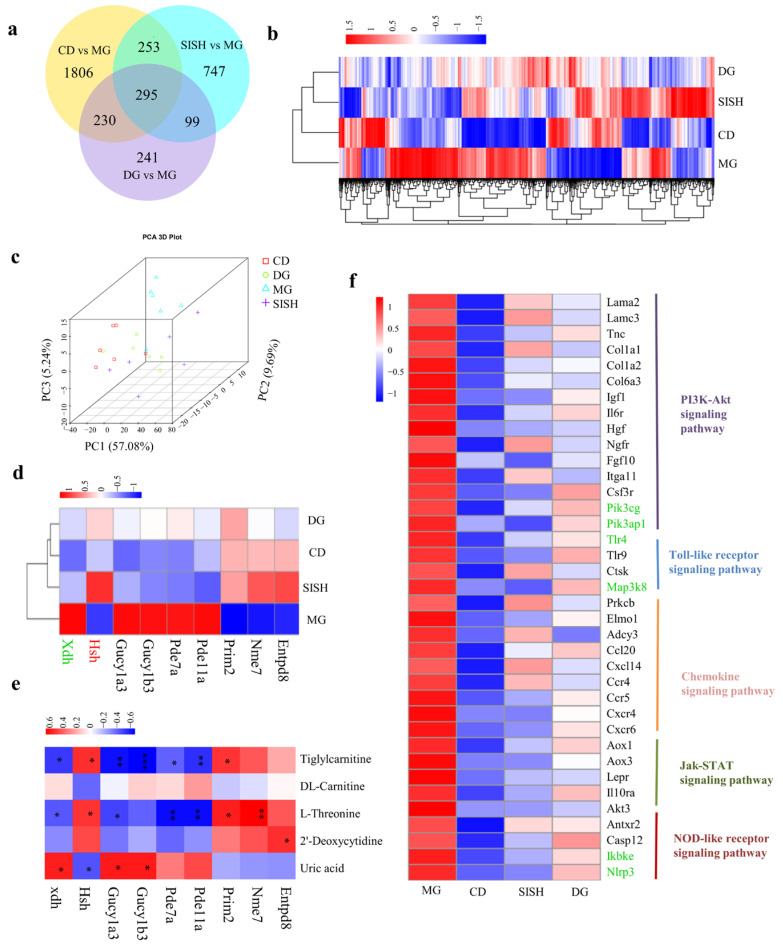
The Effects of SISH on the renal transcriptome in hyperuricemic rats. (**a**) Venn diagram. (**b**) Heatmap for hierarchical cluster analysis of differential expression genes (DEGs) (fold change of ≥ 1.5 or ≤ −1.5, *p* < 0.05). (**c**) The principal component analysis (PCA) score plot. (**d**) Heatmap for genes involved in purine metabolism. (**e**) Spearman correlation analysis between the metabolites and genes related to purine metabolism. Differences were assessed by ANOVA. (**f**) Heatmap for genes involved in inflammatory responses. Note: * *p* < 0.05, ** *p* < 0.01, and *** *p* < 0.01.

In order to further define the molecular determinants underlying SISH beneficial effects in hyperuricemic rats, we then explored the renal transcriptome. As compared with the CD group, 2584 genes were differentially expressed in the MG group. Furthermore, a total of 1394 genes were differentially expressed between the SISH and the MG groups (Figure 5a). Hierarchical clustering and PCA analysis revealed that DEGs in the SISH group were obviously distinct from those in the MG group (Figure 5b,c).

Next, we evaluated the expression of several crucial genes related to purine metabolism (Figure 5d, Supplementary Table S2). Compared with the CD group, Xdh, Gucy1α3, Gucy1β3, Pde7a, and Pde11a transcripts were markedly upregulated in the MG group, while Prim2, Nme7, and Entpd8 were substantially downregulated. Importantly, compared with the MG group, the expression levels of Xdh, Gucy1α3, Gucy1β3, Pde7a, and Pde11a were all downregulated, while Hsh was upregulated upon SISH treatment. Consistently, based on the Spearman correlation analysis, we found that the metabolite of UA was positively (*p* < 0.05) correlated with Xdh, Gucy1α3, and Gucy1β3, but negatively (*p* < 0.05) correlated with Hsh (Figure 5e). These results demonstrated that SISH could reduce UA levels by regulating genes functioning in purine metabolism.

Excessive levels of serum UA are known to increase the risk of kidney inflammation and damage [36]. In accordance, the transcriptomic analysis identified enrichment in a group of DEGs relevant to inflammation (Figure 5f). Compared with the MG group, the SISH group substantially reduced the expression of genes involved in the PI3K-Akt signaling pathway, Jak-STAT signaling pathway, Toll-like receptor signaling pathway, and NOD-like receptor signaling pathway. Among them, Tlr4, Map3k8, Ikbke, Nlrp3, Pik3cg, Pik3ap1, and Akt3 genes were significantly downregulated in the SISH group as compared with the MG group. According to GO analysis (Supplementary Figures S3 and S4), the significantly upregulated DEGs in the SISH group (as compared to the MG group) were functionally enriched in the transport and metabolic-related processes. In comparison, most of the significantly downregulated genes were associated with inflammatory responses, such as chemotaxis, ERK1 and ERK2 cascade, regulation of ERK1 and ERK2 cascade, and positive regulation of MAPK cascade. Together, these findings specified these inflammation-related genes that could mediate the beneficial effects of SISH on hyperuricemia. 

## 4. Discussion

With the increasing prevalence of hyperuricemia worldwide, it is imperative to seek more effective alternatives for hyperuricemia intervention with fewer side effects. Plant protein hydrolysates are among the best options in terms of scientific, economic, social, environmental, and industrial values. In this study, we evidenced that sacha inchi oil press-cake is a good source of bioactive protein hydrolysates that have favorable anti-hyperuricemia effects. Instead of discarding the sacha inchi oil press-cake as industrial by-products and environmental biohazards, we could obtain SISH from it by hydrolyzing proteins. A series of tests in biochemical, biological, and physiological settings helped us to define the composition and anti-hyperuricemic effects of SISH, potentializing its use and application in various food and bio-industrial domains.

Hyperuricemia is characterized by high levels of UA in serum [37], and previous studies have shown that some small molecular weight peptides (<1 kDa) could lower the serum UA level [18,38,39]. In this study, SISH was confirmed to contain a large number of small peptides (up to 90.7%, <1 kDa) and shown to reduce serum UA levels in hyperuricemic rats, effects that were validated in serum metabolome analysis, as well. Purine metabolism disorders could also lead to an increase in serum UA, in which the XOD plays a central role by catalyzing the oxidation and conversion of hypoxanthine to UA [18]. Consistently, our study found that SISH inhibited the XOD activity in serum and liver in hyperuricemic rats, providing a mechanistic explanation. It should be noted that Trp residue in peptides, as identified in the SISH oligopeptides, acts as a critical factor for achieving XOD inhibitory activity [37]. Moreover, a mixture of peptides in the SISH would confer combinatory effects on UA metabolism, likely due to synergistic interactions among these peptides [40]. In line with these findings, renal transcriptome analysis indicated that SISH could downregulate genes relevant to UA production (such as Xdh, Gucy1α3, Gucy1β3, Pde7a, and Pde11a) [41] and UA catabolism (like Hsd) that promotes the degradation of UA into allantoin [42]. Collectively, we propose that the anti-hyperuricemic activities of SISH are derived from its inhibitory activity on XOD and its regulation of genes engaged in UA metabolism.

The imbalance between UA production and excretion leads to hyperuricemia, and approximately 2/3 of UA is excreted through the renal route [9]; however, a high level of UA in serum could cause renal injury [43]. Therefore, normal renal function is an essential factor in ensuring the balance of UA production and excretion. Serum BUN and Cr are important indicators of renal functions, and consistently, in our study, both levels of BUN and Cr were enhanced in hyperuricemic rats. On the contrary, SISH treatment significantly reversed the increases in BUN and Cr, in agreement with the findings by Shan et al. [44]. In addition, high UA in serums could induce renal inflammation [36], and histopathological examination did show inflammatory cell infiltration in the interstitium of the kidney in hyperuricemic rats, while SISH again alleviated the inflammation. Mechanistically, renal transcriptome analysis in hyperuricemic rats further demonstrated that SISH downregulated genes relevant to inflammatory signaling, namely the PI3K-Akt signaling pathway, Toll-like receptor signaling pathway, chemokine signaling pathway, Jak-STAT signaling pathway, and NOD-like receptor signaling pathway. Of significance, SISH repressed the Tlr4 gene that functions as a UA receptor [45]. Upon Tlr4 activation by UA, its downstream signal cascade (like the IKK/IκB/NF-κB pathway) will be unleashed, ultimately leading to the induction of pro-inflammatory cytokines/mediators and the onset of tissue damage [36,46]. Together, we determined by multiple channels that SISH markedly alleviates the kidney damage bound with hyperuricemia. 

In addition to the predominant excretion of UA through the kidneys, the remaining about 1/3 of UA is excreted from the intestines [9]; importantly, UA excretion will be mainly converted to the intestine when kidneys become dysfunctional [43]. Gut microbiota, as an important component of the intestine, plays an active role in UA excretion [9,47]. In accordance, previous studies indicated that the anti-hyperuricemic effects of some oligopeptides were mediated by the gut microbiota, such as tuna meat oligopeptides [40], Val-Val-Tyr-Pro peptide [48], and *Apostichopus japonicus* oligopeptide [49]. As reported, the diversity of intestinal flora in the hyperuricemia group is generally lower than that in the normal group [50]. Indeed, the alpha diversity analysis in the current study revealed that the Shannon diversity index was significantly decreased in the hyperuricemic rats, whereas it was substantially enhanced by SISH supplementation. Short-chain fatty acids (SCFAs), especially butyric acid, can relieve hyperuricemia by providing energy to intestinal wall cells to promote UA excretion [9]. Compared with the hyperuricemic group, the SISH group had a significantly increased abundance of *Ruminococcaceae* (unidentified_*Ruminococcaceae*, *Oscillibacter*, *Ruminiclostridium*, *Intestinimonas*) which are considered butyrate-producing prebiotics [51]. In addition, SISH also increased the abundance of probiotics (*Akkermansia*, *Alistipes*) and reduced the population of the XOD-producing bacteria (*Streptococcus*) [10]. *Lactobacillus* is known to sequentially degrade UA to urea by synthesizing uricase, allantoinase, and allantoicase, thereby improving UA excretion [9]. Unexpectedly, we found that the relative abundance of *Lactobacillus* was higher in the MG group as compared with the CD group, and SISH decreased *Lactobacillus* in hyperuricemic rats, which is in contradiction to some reports [9,50]. We speculated that the increase in *Lactobacillus* in the MG group could reflect the body’s intrinsic compensatory system as a part of feedback at certain time points to consume excess UA in hyperuricemic rats [43]. Overall, these results clearly indicate that SISH supplementation could reverse gut microbiota dysbiosis in hyperuricemic rats.

By integrating the results of 16S rRNA gene sequencing, serum metabolome, and renal transcriptome analyses, we also found that UA level was negatively correlated with the *Ruminococcaceae* (unidentified_*Ruminococcaceae*, *Oscillibacter*, *Ruminiclostridium*, *Intestinimonas*) abundance and *Hsh* gene expression level, and an increase in those bacteria and the *Hsh* gene can enhance UA excretion [51]. In addition, UA levels positively correlated with *Streptococcus* abundance and *Xdh* gene expression, and increases in those bacteria and the *Xdh* gene would enhance UA production [10,41]. Consistently, SISH boosted the *Ruminococcaceae* abundance and Hsh gene expression and reduced the *Streptococcus* abundance and *Xdh* gene expression, consequentially leading to a decline in serum UA and relief of hyperuricemia. In summary, SISH attenuated hyperuricemia by ameliorating renal damage and maintaining intestinal flora integrity. Our findings provide new insights on the utilization of sacha inchi oil press-cake and the potential of derived SISH as a hyperuricemia therapeutic regime. 

## 5. Conclusions

In conclusion, SISH could reduce the serum UA level and improve hyperuricemia. The underlying mechanisms could be related to the inhibition of the XOD activity, regulation of gut microbiota structure, and attenuation of the renal injury in SD rats. However, additional studies are required to investigate the deeper mechanisms underlying the anti-hyperuricemic activity of SISH; for instance, fecal microbiota transplantation could be used to further explore whether alterations in the gut microbiota are a key factor in the anti-hyperuricemic activity of SISH. Overall, these results suggest that the protein in sacha inchi oil pressed-cake is a valuable source of hydrolysates, and SISH could be used as a potential functional component for preventing hyperuricemia.

## Figures and Tables

**Table 1:** The molecular weight distribution and amino acid composition of SISH.

		SISH
Molecular weight distribution (%)		
	<1 kDa	90.7
	1~2 kDa	6.0
	2~5 kDa	1.9
	>5 kDa	1.4
Amino acid composition (%)		
	Glutamate	16.7
	Aspartic acid	12.6
	Arginine	10.8
	Glycine	6.6
	Serine	5.9
	Lysine	4.9
	Valine	5.8
	Threonine	5.0
	Proline	4.1
	Leucine	7.1
	Alanine	3.9
	Tyrosine	4.4
	Isoleucine	4.7
	Phenylalanine	2.2
	Histidine	2.2
	Cysteine	2.4
	Methionine	1.0
	Essential amino acid content	30.7
	Hydrophobic amino acid	24.9

**Table 2:** The peptides sequence identified in SISH by HPLC/MS-MS analysis.

Peptide Sequence	Mass-to-Charge Ratio	Retention Time	Relative Intensity (%)	Peptide Score
TGGWSPLK	423.2326	19.9	1.19	98
WKPW	308.6679	27.18	1.55	98
FLTMEPR	447.2341	19.97	5.29	97
VVLDVK	672.4325	16.53	4.1	97
KVVL	458.3361	21.18	7.86	97
MVVKK	302.6903	21.15	8.66	97
LTGLNKL	379.7451	20.72	3.76	97
RLLVWELER	607.3524	18.13	8.13	97
KLSLEWWLK	601.8453	17.98	2.9	96
FVKLL	310.2144	25.61	5.04	96
LGDLGTKL	408.7478	22.05	1.99	96
LTGLDKL	380.2367	22.14	3.34	96
LFAEMDK	427.2123	18.29	4.69	96
EADGTLR	381.1941	14.02	1.11	96
VVLFK	303.2065	19.5	2.1	95
T(+42.01)LLNPR	378.2258	17.03	1.04	95
AYLTGLK	383.2322	18.84	1.01	95
WLPDVK	379.2173	21.21	7.68	95
VLWLPR	392.2499	30.21	1.25	95
RWQVWEDR	587.7963	21.99	2.107	95
TVLLPR	349.7339	18.2	8.63	95
LVRFPK	380.2433	20.96	1.6	95
TLLFGDK	397.2278	22.5	1.56	95
WSELVK	381.2155	18.68	1.66	95

## Data Availability

The data presented in this study are available on request from the corresponding author.

## Supplementary Materials

The following supporting information can be downloaded at: https://www.mdpi.com/article/10.3390/foods11162534/s1, Table S1, Identification of the potential biomarkers in SISH treated hyperuricemic rats; Table S2, Identification of candidate functional genes in SISH treated hyperuricemic rats; Figure S1, The serum UA levels in rats upon adenine and potassium oxonate treatments for 10 days; Figure S2, Comparison of indicated specific taxa between the four test groups; Figure S3, GO enrichment analyses between the CD and the MG groups; Figure S4, GO enrichment analyses between the SISH and the MG groups.

## References

[1] Guo Z., Zhang J.C., Wang Z.L., Ang K.Y., Huang S., Hou Q.C., Su X.Q., Qiao J.M., Zheng Y., Wang L.F. (2016). Intestinal Microbiota Distinguish Gout Patients from Healthy Humans. Sci. Rep..

[2] Zhang R., Zhan S.Y., Li S.Y., Zhu Z.Z., He J.R., Lorenzo J.M., Barba F.J. (2018). Anti-hyperuricemic and nephroprotective effects of extracts from Chaenomeles sinensis (Thouin) Koehne in hyperuricemic mice. Food Funct..

[3] Li X.Q., Gao X.X., Zhang H., Liu Y.Y., Sarker M.M.R., Wu Y.J., Chen X.H., Zhao C. (2021). The anti-hyperuricemic effects of green alga Enteromorpha prolifera polysaccharide via regulation of the uric acid transporters in vivo. Food Chem. Toxicol..

[4] Mehmood A., Zhao L., Ishaq M., Xin W., Zhao L., Wang C.T., Hossen I., Zhang H.M., Lian Y.H., Xu M.L. (2020). Anti-hyperuricemic potential of stevia (Stevia rebaudiana Bertoni) residue extract in hyperuricemic mice. Food Funct..

[5] Abeles A.M. (2015). Hyperuricemia, gout, and cardiovascular disease: An update. Curr. Rheumatol. Rep..

[6] Ma Z.M., Wang Y.F., Xu C.N., Ai F.L., Huang L., Wang J.P., Peng J., Zhou Y.M., Yin M.H., Zhang S. (2019). Obesity-Related Genetic Variants and Hyperuricemia Risk in Chinese Men. Front. Endocrinol..

[7] Grassi D., Desideri G., Di Giacomantonio A.V., Di Giosia P., Ferri C. (2014). Hyperuricemia and cardiovascular risk. High Blood Press. Cardiovasc. Prev..

[8] Su H.Y., Yang C., Liang D., Liu H.F. (2020). Research Advances in the Mechanisms of Hyperuricemia-Induced Renal Injury. BioMed Res. Int..

[9] Wang J., Chen Y., Zhong H., Chen F., Regenstein J., Hu X.S., Cai L.Y., Feng F.Q. (2021). The gut microbiota as a target to control hyperuricemia pathogenesis: Potential mechanisms and therapeutic strategies. Crit. Rev. Food Sci. Nutr..

[10] Gao Y., Sun J., Zhang Y., Shao T.J., Li H.C., Wang M.J., Zhang L., Bian H., Wen C.P., Xie Z.J. (2020). Effect of a Traditional Chinese Medicine Formula (CoTOL) on Serum Uric Acid and Intestinal Flora in Obese Hyperuricemic Mice Inoculated with Intestinal Bacteria. Evid. Based Complementary Altern. Med..

[11] Dalbeth N., Merriman T.R., Stamp L.K. (2016). Gout. Lancet.

[12] Kohagura K., Tana T., Higa A., Yamazato M., Ishida A., Nagahama K., Sakima A., Iseki K., Ohya Y. (2016). Effects of xanthine oxidase inhibitors on renal function and blood pressure in hypertensive patients with hyperuricemia. Hypertens. Res..

[13] Bailén R., González Senac N.M., López M.M., Luisa Llena M., Migoya M., Teresa Rodriguez M., de Miguel E., Torres R.J., Puig J.G. (2014). Efficacy and Safety of a Urate Lowering Regimen in Primary Gout. Nucleos. Nucleot. Nucl..

[14] Abd El-Maksoud A.A., Korany R.M.S., Abd El-Ghany I.H., El-Beltagi S.H., de Gouveia G.M.A.F. (2020). Dietary solutions to dyslipidemia: Milk protein–polysaccharide conjugates as liver biochemical enhancers. J. Food Biochem..

[15] Qi X.F., Chen H.R., Guan K.F., Wang R.C., Ma Y. (2021). Anti-hyperuricemic and nephroprotective effects of whey protein hydrolysate in potassium oxonate induced hyperuricemic rats. J. Sci. Food Agric..

[16] Li Y.J., Kang X.Y., Li Q.Y., Shi C.C., Lian Y.Y., Yuan E.D., Zhou M., Ren J.Y. (2018). Anti-hyperuricemic peptides derived from bonito hydrolysates based on in vivo hyperuricemic model and in vitro xanthine oxidase inhibitory activity. Peptides.

[17] Wu Y.Q., He H., Hou T. (2021). Purification, identification, and computational analysis of xanthine oxidase inhibitory peptides from kidney bean. J. Food Sci..

[18] He W.W., Su G.W., Sun-Waterhouse D., Waterhouse G.I.N., Zhao M.M., Liu Y. (2019). In vivo anti-hyperuricemic and xanthine oxidase inhibitory properties of tuna protein hydrolysates and its isolated fractions. Food Chem..

[19] Li Q., Kang X., Shi C., Li Y., Majumder K., Ning Z., Ren J. (2018). Moderation of hyperuricemia in rats via consuming walnut protein hydrolysate diet and identification of new antihyperuricemic peptides. Food Funct..

[20] Chirinos R., Zuloeta G., Pedreschi R., Mignolet E., Larondelle Y., Campos D. (2013). Sacha inchi (*Plukenetia volubilis*): A seed source of polyunsaturated fatty acids, tocopherols, phytosterols, phenolic compounds and antioxidant capacity. Food Chem..

[21] Torres Sánchez E.G., Hernández-Ledesma B., Gutiérrez L.F. (2021). Sacha Inchi Oil Press-cake: Physicochemical Characteristics, Food-related Applications and Biological Activity. Food Rev. Int..

[22] Chirinos R., Aquino M., Pedreschi R., Campos D. (2017). Optimized Methodology for Alkaline and Enzyme-Assisted Extraction of Protein from Sacha Inchi (*Plukenetia volubilis*) Kernel Cake. J. Food Process. Eng..

[23] Lai X., Pan S., Zhang W., Sun L., Li Q., Chen R., Sun S. (2022). Properties of ACE inhibitory peptide prepared from protein in green tea residue and evaluation of its anti-hypertensive activity. Process. Biochem..

[24] Chirinos R., Pedreschi R., Campos D. (2020). Enzyme-assisted hydrolysates from sacha inchi (*Plukenetia volubilis*) protein with in vitro antioxidant and antihypertensive properties. J. Food Process. Preserv..

[25] Rawdkuen S., Rodzi N., Pinijsuwan S. (2018). Characterization of sacha inchi protein hydrolysates produced by crude papain and Calotropis proteases. Lwt-Food Sci. Technol..

[26] Perez-Galvez R., Morales-Medina R., Espejo-Carpio F., Guadix A., Guadix E.M. (2016). Modelling of the production of ACE inhibitory hydrolysates of horse mackerel using proteases mixtures. Food Funct..

[27] Benjakul S., Oungbho K., Visessanguan W., Thiansilakul Y., Roytrakul S. (2009). Characteristics of gelatin from the skins of bigeye snapper, Priacanthus tayenus and Priacanthus macracanthus. Food Chem..

[28] Li G.S., Zhan J.Q., Hu L.P., Yuan C.H., Takaki K., Ying X.G., Hu Y.Q. (2021). Identification of a new antioxidant peptide from porcine plasma by in vitro digestion and its cytoprotective effect on H_2_O_2_ induced HepG2 model. J. Funct. Foods.

[29] Liao W.W., Chen H., Jin W.G., Yang Z.N., Cao Y., Miao J.Y. (2020). Three Newly Isolated Calcium-Chelating Peptides from Tilapia Bone Collagen Hydrolysate Enhance Calcium Absorption Activity in Intestinal Caco-2 Cells. J. Agric. Food Chem..

[30] Morgan X.C., Tickle T.L., Sokol H., Gevers D., Devaney K.L., Ward D.V., Reyes J.A., Shah S.A., LeLeiko N., Snapper S.B. (2012). Dysfunction of the intestinal microbiome in inflammatory bowel disease and treatment. Genome Biol..

[31] Kanehisa M., Goto S., Sato Y., Furumichi M., Tanabe M. (2012). KEGG for integration and interpretation of large-scale molecular data sets. Nucleic Acids Res..

[32] Chen G., Tan M.L., Li K.K., Leung P.C., Ko C.H. (2015). Green tea polyphenols decreases uric acid level through xanthine oxidase and renal urate transporters in hyperuricemic mice. J. Ethnopharmacol..

[33] Tian B.M., Zhao J.H., Zhang M., Chen Z.F., Ma Q.Y., Liu H.C., Nie C.X., Zhang Z.Q., An W., Li J.X. (2021). Lycium ruthenicum Anthocyanins Attenuate High-Fat Diet-Induced Colonic Barrier Dysfunction and Inflammation in Mice by Modulating the Gut Microbiota. Mol. Nutr. Food Res..

[34] Wang Y.N., Zhao M., Xin Y., Liu J.J., Wang M., Zhao C.J. (2016). ^1^H NMR and MS based metabolomics study of the therapeutic effect of Cortex Fraxini on hyperuricemic rats. J. Ethnopharmacol..

[35] Wang C., Pan Y., Zhang Q.Y., Wang F.M., Kong L.D. (2012). Quercetin and allopurinol ameliorate kidney injury in STZ-treated rats with regulation of renal NLRP3 inflammasome activation and lipid accumulation. PLoS ONE.

[36] Zhou Y., Fang L., Jiang L., Wen P., Cao H.D., He W.C., Dai C.S., Yang J.W. (2012). Uric acid induces renal inflammation via activating tubular NF-kappaB signaling pathway. PLoS ONE.

[37] Li Q.Y., Shi C.C., Wang M., Zhou M., Liang M., Zhang T., Yuan E.D., Wang Z., Yao M.J., Ren J.Y. (2019). Tryptophan residue enhances in vitro walnut protein-derived peptides exerting xanthine oxidase inhibition and antioxidant activities. J. Funct. Foods.

[38] Liu N.X., Wang Y., Zeng L., Yin S.G., Hu Y., Li S.S., Fu Y., Zhang X.P., Xie C., Shu L.J. (2020). RDP3, A Novel Antigout Peptide Derived from Water Extract of Rice. J. Agric. Food Chem..

[39] Wei L.Y., Ji H.W., Song W.K., Peng S., Zhan S.H., Qu Y.S., Chen M., Zhang D., Liu S.C. (2021). Hypouricemic, hepatoprotective and nephroprotective roles of oligopeptides derived from Auxis thazard protein in hyperuricemic mice. Food Funct..

[40] Han J.J., Wang X.F., Tang S.S., Lu C.Y., Wan H.T., Zhou J., Li Y., Ming T.H., Wang Z.J., Su X.R. (2020). Protective effects of tuna meat oligopeptides (TMOP) supplementation on hyperuricemia and associated renal inflammation mediated by gut microbiota. FASEB J..

[41] Wang C.H., Zhang C., Xing X.H. (2016). Xanthine dehydrogenase: An old enzyme with new knowledge and prospects. Bioengineered.

[42] French J.B., Ealick S.E. (2011). Structural and kinetic insights into the mechanism of 5-hydroxyisourate hydrolase from Klebsiella pneumoniae. Acta. Crystallogr. D.

[43] Pan J., Shi M., Guo F., Ma L., Fu P. (2021). Pharmacologic inhibiting STAT3 delays the progression of kidney fibrosis in hyperuricemia-induced chronic kidney disease. Life Sci..

[44] Shan B., Chen T., Huang B.X., Liu Y., Chen J. (2021). Untargeted metabolomics reveal the therapeutic effects of Ermiao wan categorized formulas on rats with hyperuricemia. J. Ethnopharmacol..

[45] Ru L.B., Scott P., Sydlaske A., Rose D.M., Terkeltaub R. (2005). Innate immunity conferred by Toll-like receptors 2 and 4 and myeloid differentiation factor 88 expression is pivotal to monosodium urate monohydrate crystal-induced inflammation. Arthritis Rheumatol..

[46] Bi F.F., Chen F., Li Y.N., Wei A., Cao W.S. (2018). Klotho preservation by Rhein promotes toll-like receptor 4 proteolysis and attenuates lipopolysaccharide-induced acute kidney injury. J. Mol. Med..

[47] Mehmood A., Zhao L., Wang C.T., Hossen I., Raka R.N., Zhang H.M. (2019). Stevia residue extract increases intestinal uric acid excretion via interactions with intestinal urate transporters in hyperuricemic mice. Food Funct..

[48] Xie X.S., Zhang L., Yuan S., Li H.L., Zheng C.J., Xie S.S., Sun Y.B., Zhang C.H., Wang R.K., Jin Y. (2021). Val-Val-Tyr-Pro protects against non-alcoholic steatohepatitis in mice by modulating the gut microbiota and gut-liver axis activation. J. Cell. Mol. Med..

[49] Lu C.Y., Tang S.S., Han J.J., Fan S.Q., Huang Y.M., Zhang Z., Zhou J., Ming T.H., Li Y., Su X.R. (2021). Apostichopus japonicus Oligopeptide Induced Heterogeneity in the Gastrointestinal Tract Microbiota and Alleviated Hyperuricemia in a Microbiota-Dependent Manner. Mol. Nutr. Food Res..

[50] Yu Y.R., Liu Q.P., Li H.C., Wen C.P., He Z.X. (2018). Alterations of the Gut Microbiome Associated With the Treatment of Hyperuricaemia in Male Rats. Front. Microbiol..

[51] Solano-Aguilar G.I., Jang S., Lakshman S., Gupta R., Beshah E., Sikaroodi M., Vinyard B., Molokin A., Gillevet P.M., Urban J.F. (2018). The Effect of Dietary Mushroom Agaricus bisporus on Intestinal Microbiota Composition and Host Immunological Function. Nutrients.

