# Supplementary material for: Sacha Inchi Oil Press-Cake Protein Hydrolysates Exhibit Anti-Hyperuricemic Activity via Attenuating Renal Damage and Regulating Gut Microbiota
Source: Foods. 2022 Aug 22;11(16):2534. doi: 10.3390/foods11162534 (PMC9407120; doi:10.3390/foods11162534)
Supplement: Supplementary file 1 [file foods-11-02534-s001.zip › foods-1853121-supplementary.pdf]

Supplementary Information

Sacha Inchi Oil Press-Cake Protein Hydrolysates Exhibit Anti-Hyperuricemic Activity via Attenuating Renal Damage and Regulating Gut Microbiota

Kun Wang, Shanshan Wu, Pan Li, Nan Xiao, Jiamin Wen, Jinming Lin, Siming Lu, Xin Cai, Yanan Xu and Bing Du *

College of Food Science, South China Agricultural University, Guangzhou 510642, China

* Correspondence: dubing@scau.edu.cn

Contents

Table S1. Identification results of potential biomarkers of SISH treated hyperuricemic rats.

Table S2. Identification results of potential genes of SISH treated hyperuricemic rats.

Figure S1. The serum UA levels in rats after adenine and potassium oxonate treatment for 10 days.

Figure S2. The comparison of several specific taxa between the four groups.

Figure S3. GO enrichment analysis between the CD and MG group.

Figure S4. GO enrichment analysis between the SISH and MG group

Table S1. Identification results of potential biomarkers of SISH treated hyperuricemic rats.

Name	Formula	Molecular Weight	RT [min]	CD vs MG		SISH vs MG		DG vs MG	
				log2FC	*P* value	log2FC	*P* value	log2FC	*P* value
Uric acid	$C_5H_4N_4O_3$	168.0286	1.39	-0.56334	0.017451	-0.79565	0.00818	-0.49106	0.039769
2'-Deoxycytidine	$C_9H_{13}N_3O_4$	227.0908	1.375	0.340359	0.013731	0.207622	0.038931	0.208908	0.136709
L-Threonine	$C_4H_9NO_3$	119.0584	1.317	0.66064	0.002534	0.570697	0.030766	0.475711	0.002168
DL-Carnitine	$C_7H_{15}NO_3$	161.1054	1.308	0.771147	3.28E-05	0.207622	0.038931	0.208908	0.136709
Tiglylcarnitine	$C_{12}H_{21}NO_4$	243.1474	10.543	4.342748	0.003476	4.364824	0.00036	4.480659	0.000111
Oleic acid	$C_{18}H_{34}O_2$	282.2563	13.165	-0.85889	0.010961	-0.37712	0.093781	-0.97005	0.00355
D-Sphingosine	$C_{18}H_{37}NO_2$	299.2827	13.036	-0.66207	0.005482	-0.36393	0.117863	-0.62886	0.00207

Table S2. Identification results of potential genes of SISH treated hyperuricemic rats.

		Genen ID	Gene_description	FPKM				*p* value		
				CD	MG	TS	DG	CD vs MG	SISH vs MG	DG vs MG
**Purine metabolism**	Xdh	497811	xanthine dehydrogenase	23.45382	41.46954	26.4438	27.42506	1.35E-11	0.00013	6.13E-05
	Hsh	293613	5-hydroxyisourate hydrolase	0.85316	0.457308	1.891492	1.200592	0.048056	3.69E-05	0.001063
	Gucy1α3	497757	guanylate cyclase 1 soluble subunit alpha 3%2C transcript variant X3	2.550802	6.251409	3.063274	3.556875	2.79E-05	0.004153	0.01219
	Gucy1β3	25202	guanylate cyclase 1 soluble subunit beta 3	2.144531	4.554913	2.149856	2.832124	0.000207	0.000398	0.022819
	Pde7a	81744	phosphodiesterase 7A	0.84849	1.488257	0.843783	1.085454	0.000775	0.005719	0.080955

	Pde11a	140928	phosphodiesterase 11A%2C transcript variant 3	0.339043	0.524972	0.285331	0.364973	0.020632	0.011814	0.024277
	Prim2	301323	primase%2C DNA%2C polypeptide 2%2C transcript variant X3	3.332274	2.151712	3.421895	3.389925	0.019147	0.008924	0.041126
	Nme7	171566	NME/NM23 family member 7	15.39423	9.511376	17.96419	13.75155	0.003143	0.000396	0.025182
	Entpd8	613267	ectonucleoside triphosphate diphosphohydrolase 8%2C transcript variant X2	1.072657	0.52895	1.322952	0.841691	0.044028	0.011881	0.161162
**PI3K-Akt signaling pathway**	Lama2	309368	laminin subunit alpha 2%2C transcript variant X1	0.67335	1.862958	1.40216	1.174938	7.76E-10	0.072674	0.027227
	Lamc3	311862	laminin subunit gamma 3	1.330097	4.420984	3.740406	2.499518	3.68E-07	0.520866	0.013937
	Tnc	116640	tenascin C%2C transcript variant X2	0.369395	1.160301	0.581021	0.752895	1.52E-05	0.01544	0.087236

Col1a1	29393	collagen%2C type I%2C alpha 1	3.008136	7.509262	6.127585	4.466024	0.000333	0.384752	0.03269
Col1a2	84352	collagen type I alpha 2 chain	1.434895	4.491017	2.238209	2.45018	5.86E-05	0.096356	0.045279
Col6a3	367313	collagen type VI alpha 3 chain%2C transcript variant X3	2.004719	5.196997	2.9723	2.779848	1.33E-06	0.00169	0.003658
Igf1	24482	insulin-like growth factor 1%2C transcript variant X2	1.270585	2.173341	1.322244	1.615256	0.000166	0.01114	0.018973
Il6r	24499	interleukin 6 receptor%2C transcript variant X3	0.482404	1.180346	0.725067	0.867891	0.000514	0.022826	0.124558
Hgf	24446	hepatocyte growth factor%2C transcript variant X1	0.52943	2.186394	0.640202	0.751581	0.000519	0.042185	0.013426
Ngfr	24596	nerve growth factor receptor	0.124697	0.343179	0.303448	0.219767	0.000919	0.680736	0.019986

	Fgf10	25443	fibroblast growth factor 10	1.427312	2.950082	1.054515	1.551883	0.001153	7.78E-05	0.027459
	Itga11	315744	integrin subunit alpha 11%2C transcript variant X2	0.063095	0.266051	0.183434	0.122884	0.001534	0.308111	0.043345
	Csf3r	298518	colony stimulating factor 3 receptor%2C transcript variant X7	0.114878	0.237507	0.126512	0.201489	0.003292	0.042175	0.470152
	Pik3cg	298947	phosphatidylinositol-4%2C5-bisphosphate 3-kinase%2C catalytic subunit gamma%2C transcript variant X4	0.394424	1.012127	0.637459	0.802559	7.43E-05	0.047538	0.240065
	Pik3ap1	294048	phosphoinositide-3-kinase adaptor protein 1%2C transcript variant X2	3.742369	5.91159	3.227079	4.562143	0.040961	0.038183	0.284879
**Toll-like receptor signaling pathway**	TLR4	29260	toll-like receptor 4	0.519254	1.488081	0.811838	0.984717	5.17E-08	0.00234	0.013284
	TLR9	338457	toll-like receptor 9	0.064583	0.400321	0.104111	0.275298	0.000942	0.039877	0.436613

	Ctsk	29175	cathepsin K	6.283218	14.88338	12.54954	9.473261	0.005412	0.448697	0.026934
	Map3k8	116596	mitogen-activated protein kinase kinase kinase 8	0.873254	1.506402	0.798035	1.210414	0.0085	0.013808	0.332358
**Chemokine signaling pathway**	Prkcb	25023	protein kinase C%2C beta%2C transcript variant X1	0.449119	0.991597	0.915111	0.700788	8.56E-05	0.702674	0.035259
	Elmo1	361251	engulfment and cell motility 1	0.115169	0.237926	0.134894	0.166798	0.001202	0.009807	0.040123
	Adcy3	64508	adenylate cyclase 3	0.720147	1.269672	1.048119	0.747518	0.001921	0.23919	0.001883
	Ccl20	29538	C-C motif chemokine ligand 20%2C transcript variant X1	1.272579	3.968931	2.337615	2.839513	8.48E-06	0.014034	0.318103
	Cxcl14	306748	C-X-C motif chemokine ligand 14	0.63043	2.872617	2.353839	1.502387	0.000615	0.518419	0.013252

	Ccr4	171054	C-C motif chemokine receptor 4%2C transcript variant X1	0.054409	0.299426	0.219696	0.154245	0.008624	0.364936	0.040034
	Ccr5	117029	chemokine (C-C motif) receptor 5%2C transcript variant X1	0.417682	1.565365	0.610788	0.870348	0.000193	0.046951	0.06277
	Cxcr4	60628	C-X-C motif chemokine receptor 4	2.046051	3.691624	2.018142	2.529716	0.031424	0.002021	0.053425
	Cxcr6	1E+08	C-X-C motif chemokine receptor 6	0.754925	2.371866	0.877056	1.376069	0.033403	0.035149	0.242439
**Jak-STAT signaling pathway**	Aox1	54349	aldehyde oxidase 1%2C transcript variant X1	0.372369	1.580103	0.707232	1.00384	6.79E-09	0.001617	0.136667
	Aox3	493909	aldehyde oxidase 3	0.074322	0.314513	0.080106	0.15076	0.000113	0.001185	0.041306
	Lepr	24536	leptin receptor	0.376949	2.375132	0.648944	0.732255	0.000305	0.019113	0.009547

	Il10ra	117539	interleukin 10 receptor subunit alpha%2C transcript variant X1	0.882465	1.853247	1.09829	1.457476	0.00085	0.005148	0.179241
	Akt3	29414	AKT serine/threonine kinase 3	1.414656	2.602654	1.435131	1.560748	0.018587	0.070881	0.029536
**NOD-like receptor signaling pathway**	Antxr2	305633	anthrax toxin receptor 2%2C transcript variant X1	1.626702	3.328393	2.663078	2.596613	1.11E-05	0.054221	0.034234
	Casp12	156117	caspase 12	0.696534	2.618516	1.284813	2.186887	1.38E-05	0.028566	0.597839
	Ikbke	363984	inhibitor of kappa light polypeptide gene enhancer in B-cells%2C kinase epsilon%2C transcript variant X1	1.040737	1.957693	1.245845	1.500713	0.001063	0.007868	0.210582
	Nlrp3	287362	NLR family%2C pyrin domain containing 3%2C transcript variant X2	0.246916	0.655082	0.284121	0.477519	0.003473	0.034057	0.277912

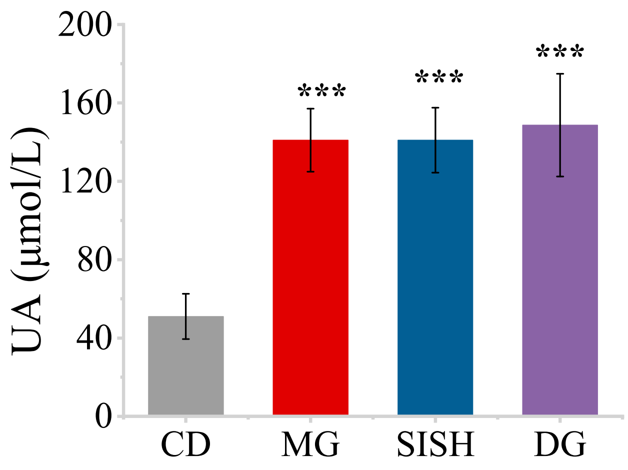

Figure S1. The serum UA levels in rats after adenine and potassium oxonate treatment for 10 days. ***$p < 0.001$ vs. CD

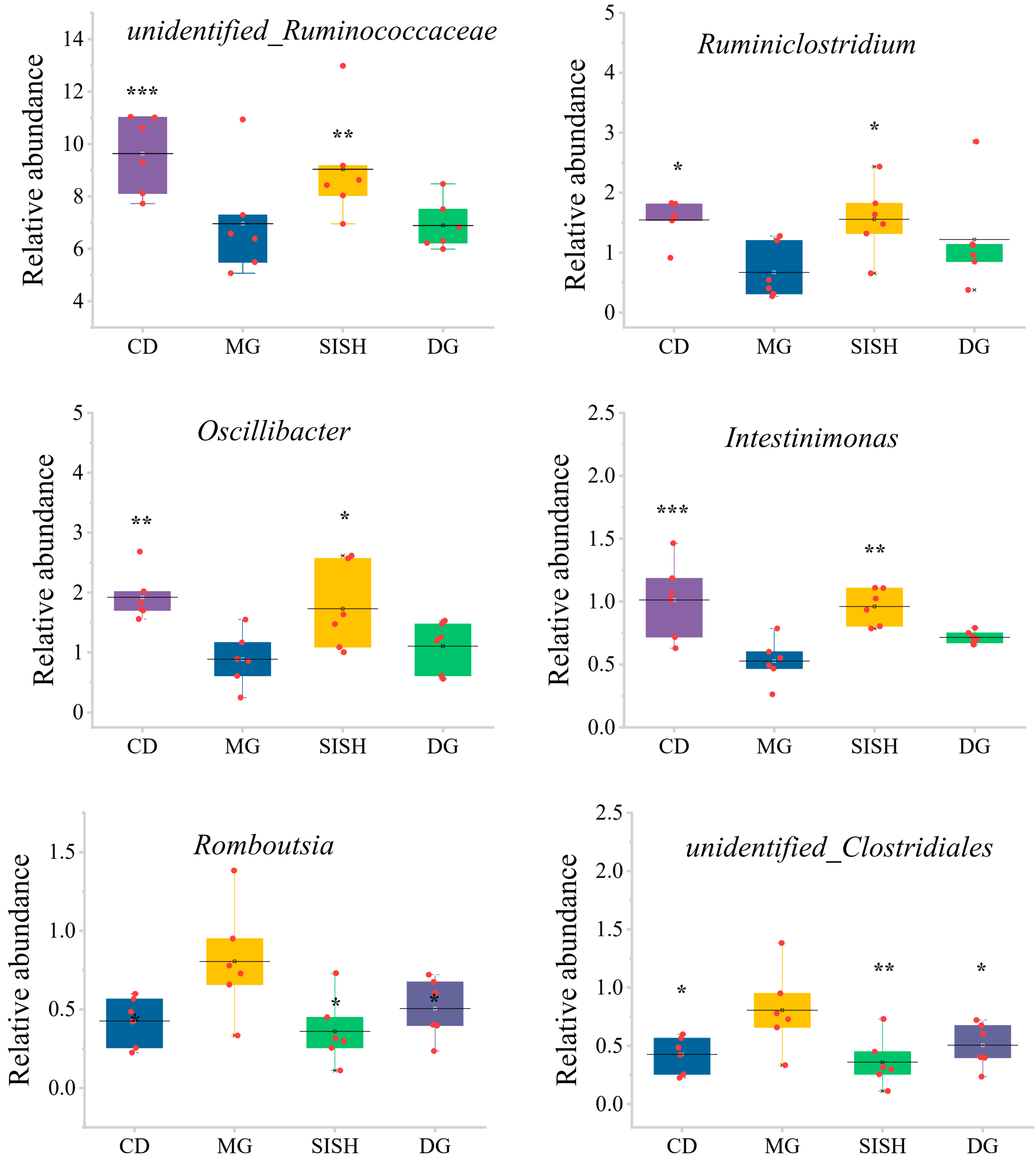

Figure S2. The comparison of several specific taxa between the four groups.

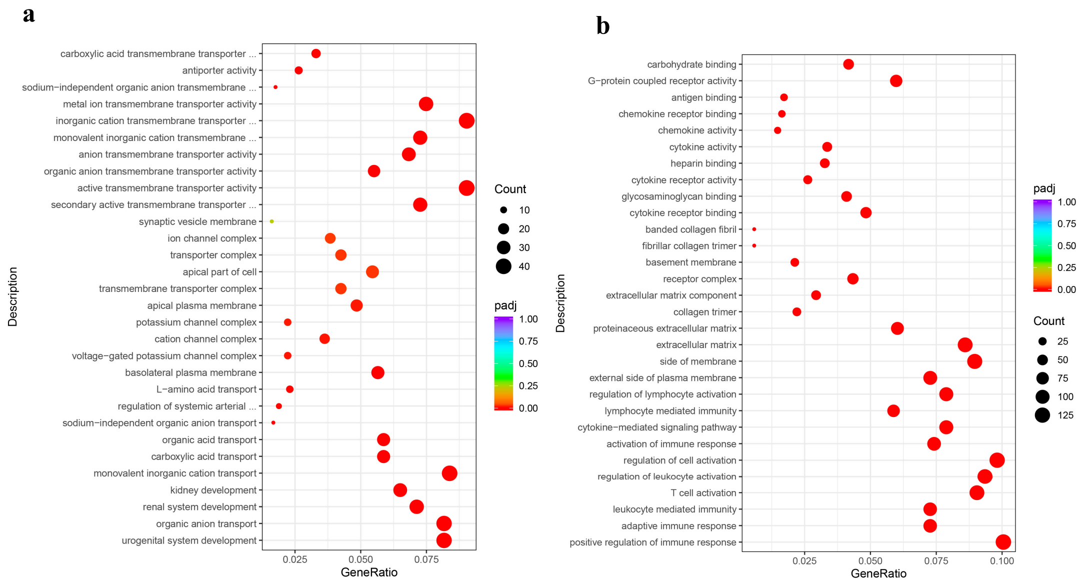

Figure S3. GO enrichment analysis between the CD and MG group.

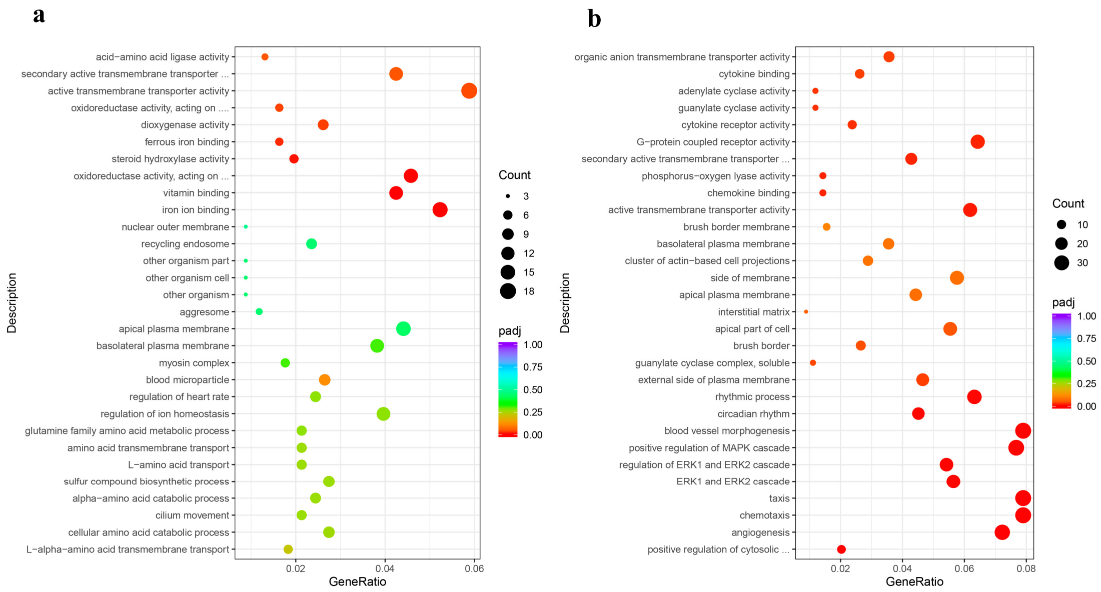

Fig. S4 GO enrichment analysis between the SISH and MG group.
